# Sagittal Plane Kinematic Deviations and Spatio-Temporal Gait Characteristics in Children with Idiopathic Toe Walking: A Comparative Analysis Using Statistical Parametric Mapping

**DOI:** 10.3390/diagnostics15050575

**Published:** 2025-02-27

**Authors:** Rocio Pozuelo-Calvo, Almudena Serrano-Garcia, Yolanda Archilla-Bonilla, Angel Ruiz-Zafra, Manuel Noguera-Garcia, Kawtar Benghazi-Akhlaki, Miguel Membrilla-Mesa, Carla DiCaudo, Jose Heredia-Jimenez

**Affiliations:** 1 Physical Medicine and Rehabilitation Department, Hospital Universitario Virgen de las Nieves, 18013 Granada, Spain; almusega@hotmail.com (A.S.-G.); archilanda1@hotmail.com (Y.A.-B.); mmembrilla@gmail.com (M.M.-M.); 2 Instituto de Investigación Biosanitaria ibs.GRANADA, 18012 Granada, Spain; dr.dicaudo@gmail.com; 3 Department of Radiology and Physical Medicine, University of Granada, 18016 Granada, Spain; 4 Department of Software Engineering, School of Computer and Telecommunication Engineering (ETSIIT), University of Granada, 18014 Granada, Spain; angelr@ugr.es (A.R.-Z.); mnoguera@ugr.es (M.N.-G.); benghazi@ugr.es (K.B.-A.); 5 Department of Physical Education & Sports, University of Granada, 18071 Granada, Spain; 6 Human Behavior & Motion Analysis Lab (HubemaLab), University of Granada, 51001 Ceuta, Spain

**Keywords:** idiopathic toe walking, gait analysis, pediatric, biomechanics, statistical parametric mapping, sagittal plane, kinematics, motion capture, pediatric locomotion disorder

## Abstract

**Background/Objectives**: Idiopathic Toe Walking (ITW) is a pediatric gait disorder characterized by persistent toe-to-heel ambulation in the absence of neurological, orthopedic, or developmental abnormalities. While spatio-temporal parameters often remain within normal ranges, subtle but clinically significant kinematic deviations may underlie compensatory mechanisms that sustain gait functionality. This study aims to evaluate spatio-temporal and sagittal plane kinematic differences between children with ITW and typically developing peers using Statistical Parametric Mapping (SPM). **Methods**: A cohort of 30 children with ITW and 30 typically developing peers aged 6–12 years participated in this study. Spatio-temporal variables, including step length, cadence, stride length, walking speed, single support phase, and swing phase, were analyzed using a three-dimensional motion capture system. Sagittal plane kinematics of the pelvis, hip, knee, and ankle were compared between groups using SPM to identify significant deviations across the gait cycle. **Results**: Significant differences were identified in the single support and swing phases, with higher values observed in the ITW group (*p* < 0.05). Sagittal plane kinematics revealed a consistent reduction in anterior pelvic tilt (*p* = 0.002), reduced hip and knee flexion during stance and swing phases (*p* < 0.001), and excessive ankle plantarflexion during early stance and terminal swing phases (*p* < 0.001). The plantarflexion observed at the end of the gait cycle corresponded to early gait phases due to methodological considerations of the coordinate-based event detection algorithm. **Conclusions**: Children with ITW demonstrate distinct spatio-temporal adaptations, including increased single support and swing phases, along with reduced walking velocity compared to typically developing peers. These findings, coupled with significant sagittal plane kinematic deviations, suggest altered neuromuscular control and joint mechanics. These insights highlight the importance of detailed kinematic analyses to identify biomechanical deficits and inform targeted interventions. Future research should explore the long-term musculoskeletal consequences of these deviations and optimize therapeutic strategies, such as physical therapy and orthotic interventions, to improve gait functionality and quality of life.

## 1. Introduction

Idiopathic Toe Walking (ITW) is a gait abnormality characterized by persistent toe-to-heel contact during ambulation in the absence of identifiable neurological, muscular, or skeletal conditions [1,2]. ITW primarily affects children aged five and older, with a reported prevalence of 5% to 12%, though its etiology remains unclear [3]. As a diagnosis of exclusion, ITW is confirmed only after ruling out other potential causes such as neurological, orthopedic, or psychiatric disorders [4,5]. Unlike other conditions associated with equinus gait patterns, children with ITW often retain the ability to partially normalize their gait, suggesting that biomechanical interventions may be effective [6].

Toe-walking is considered a normal developmental phase in children under three years of age. However, ITW is diagnosed when this behavior persists beyond this age threshold [7]. Children with ITW generally exhibit normal neurological development, adequate muscle strength, and intact selective motor control but demonstrate a marked preference for walking on the balls of their feet [8]. Prior to an ITW diagnosis, differential diagnoses—including cerebral palsy (CP), peripheral neuropathy, spinal dysraphism, and myopathies—must be thoroughly excluded [9,10]. Accurate differentiation from these conditions, many of which share overlapping gait characteristics, underscores the critical importance of precise and comprehensive gait analysis.

ITW is clinically defined by an inability to achieve heel strike during the initial contact phase of the gait cycle and the absence of full foot contact during the stance phase [11]. Neurological examinations typically reveal children with ITW to be developmentally normal, with preserved muscle strength, although they exhibit a pronounced preference for toe-walking [12]. While reduced ankle range of motion (ROM) is observed in some cases, it is not universally present [13,14]. Diagnostic tools such as electromyography (EMG), 3D motion analysis, dual-axis accelerometers, and specialized questionnaires like the “toe-walking tool” have been employed to assess ITW [15,16,17,18,19]. However, EMG has shown inconsistent reliability, limiting its diagnostic utility in this context [20,21,22]. Gait analysis has proven valuable in distinguishing ITW from CP and other conditions, though its outcomes can be affected by the heterogeneity of study populations [11,23,24].

Three-dimensional motion analysis is considered the gold standard method for the kinematic evaluation during walking in healthy and pathological populations [25]. In addition, a previous study [26] showed that the most of the parameters calculated on gait curves are more reliable on the sagittal plane with respect to the transversal and coronal planes. This instrumental methodology provides an objective, quantitative framework for describing toe-walking patterns, making it an essential tool in ITW research and diagnosis [23,27,28]. Despite its significance, studies focusing exclusively on ITW remain limited, typically involving small sample sizes (6–60 participants) and emphasizing clinical reports, family histories, ROM evaluations, and qualitative gait descriptions [13,14,15]. Previous investigations have identified excessive gastrocnemius activity, restricted ankle ROM, and impaired ankle rocker formation, which highlight the complex biomechanical adaptations associated with ITW [29].

Traditional gait analysis of Idiopathic Toe Walking (ITW) often relies on discrete variables, such as peak joint angles or moments, which provide only snapshots of movement and may fail to capture the complex, time-dependent nature of gait abnormalities. Statistical Parametric Mapping (SPM) has emerged as a powerful tool in biomechanics, enabling continuous statistical comparisons across entire kinematic and kinetic waveforms [30,31]. Unlike conventional statistical approaches—such as point-wise analysis, which evaluates specific time points (e.g., peak flexion or extension)—SPM assesses entire kinematic waveforms continuously, allowing for a more sensitive and comprehensive evaluation of gait deviations throughout the entire gait cycle. This approach reduces the risk of overlooking subtle but clinically meaningful differences. SPM has been widely validated and applied in biomechanical research, demonstrating its efficacy in detecting continuous gait differences [32,33].

The ability of SPM to analyze joint kinematics across continuous time series allows for the identification of region-specific deviations in pelvis, hip, knee, and ankle movements, providing a more detailed and statistically reliable characterization of gait deviations in children with ITW. These insights offer a deeper understanding of compensatory strategies and biomechanical alterations associated with ITW, which traditional discrete-point analysis often fails to capture [30].

Despite its advantages, SPM remains underutilized in ITW research, particularly in studies involving Spanish pediatric populations. Expanding its application in this context could refine our understanding of ITW-specific gait patterns and support the development of targeted interventions. By incorporating SPM in this study, we provide a more detailed and statistically robust characterization of gait deviations in children with ITW, identifying precise region-specific abnormalities that can inform clinical decision-making and therapeutic strategies.

This study aims to address gaps in the existing literature by employing SPM to provide a detailed characterization of gait deviations in children with ITW. Through this advanced analytical approach, we seek to enhance clinical decision-making and develop targeted interventions to improve functional outcomes and quality of life in affected children.

## 2. Materials and Methods

### 2.1. Participants

A total of sixty children participated in the study, including 30 children diagnosed with ITW (23 males; age: 10.3 ± 2.1 years; height: 1.49 ± 0.13 m; weight: 42.5 ± 11.4 kg) and 30 typically developing peers (20 males; age: 11.1 ± 1.9 years; height: 1.52 ± 0.24 m; weight: 47.31 ± 9.6 kg). Each child’s height and weight were measured using a calibrated scale and measuring rod (SECA769, Hamburg, Germany). All participants were volunteers, and their parents provided informed consent prior to participation. Children were excluded if they had recent orthopedic trauma or neurological disorders or if they were unable to walk independently. This study received ethical approval from the Andalusian Regional Government’s Department of Health and Families Ethics Committee.

### 2.2. Procedures

Parents completed a questionnaire about their child’s medical history, focusing on motor or neurological disorders, before data collection. During the experiment, children walked along a 20 m walkway at a self-selected pace while their movements were recorded using a 3D motion capture system (Qualisys AB, Gothenburg, Sweden). The calibrated anatomical systems technique (CAST) was used to track markers placed on specific anatomical landmarks [34]. Reflective markers were positioned on the first and fifth metatarsal heads, the second metatarsophalangeal joint, the medial and lateral malleoli, the posterior calcaneus, the lateral and medial femoral epicondyles, and the anterior and posterior superior iliac spines. Clusters containing four markers each were placed on the lateral thigh and shank of both legs. Retro-reflective markers (Ø14 mm) were adhered to the skin using double-sided tape, following the methodology recommended by Van Sint Jan [35].

### 2.3. Data Processing and Analysis

Motion data were recorded using Qualisys Track Manager v. 2024.1 software (QTM, Qualisys, Sweden) and processed with VISUAL 3D v.6.0 software (C-Motion Inc., Germantown, MD, USA). Marker trajectories for all trials were interpolated to a maximum gap of 10 frames using a third-order polynomial and then smoothed with a second-order Butterworth lowpass filter at 6 Hz. The global reference coordinate system defined the XYZ directions as follows: x-axis (mediolateral), y-axis (anterior/posterior), and z-axis (axial). The XYZ Cardan rotation sequence was used for joint kinematics, with the X-axis representing flexion–extension, the Y-axis representing abduction–adduction, and the Z-axis representing axial rotation.

In this study, analyses focused exclusively on the X-axis (sagittal plane) due to its clinical relevance in ITW. Sagittal plane movements are integral to the characterization of ITW, and deviations in this plane are strongly associated with the biomechanical and functional abnormalities of this gait pattern. Movements in the Y-axis (abduction–adduction) and Z-axis (axial rotation) were excluded, as they are less pertinent to the scope of this research.

Before data collection, participants completed a familiarization phase consisting of three trials on the walkway. During testing, at least nine left strides and nine right strides were recorded and averaged for each participant. A coordinate-based algorithm [36] was used to identify initial contact events, which were then employed to calculate gait cycle durations.

### 2.4. Output Variables

The following spatiotemporal variables were analyzed: walking velocity (m/s), cadence (steps/min), step length (m), and the swing phase, stance phase, single support phase, and double support phase, all expressed as percentages of the gait cycle (GC). Spatiotemporal parameters were calculated as the average of both legs and normalized to each participant’s GC duration, ensuring inter-subject comparability.

Kinematic variables in the sagittal plane were derived from joint angles at the pelvis, hip, knee, and ankle. Pelvic angles were measured relative to a global reference system, while hip, knee, and ankle angles were calculated as relative angles between adjacent segments (e.g., pelvis and femur for the hip angle). For each joint, mean and standard deviation values (in degrees) were computed and normalized across the GC (0–100%), providing a detailed analysis of joint behavior throughout the entire gait cycle.

### 2.5. Statistical Analysis

All statistical analyses were conducted using MATLAB v. R2023b (MathWorks, Natick, MA, USA) and SPSS (IBM SPSS Statistics, Version 27.0, Armonk, NY, USA). A significance level of *p* < 0.05 was adopted for all tests.

To evaluate spatiotemporal variables such as velocity, cadence, step length, and the phases of the gait cycle, independent-samples *t*-tests were performed to compare children with ITW and typically developing peers. Data assumptions of normality and homogeneity of variance were verified using the Shapiro–Wilk and Levene’s tests, respectively.

Kinematic data, limited to the sagittal plane (X-axis) for its biomechanical relevance in ITW, were analyzed using Statistical Parametric Mapping (SPM-1D). SPM was applied to compare continuous joint angle waveforms across the entire GC (0–100%) for both groups. Unlike traditional discrete point analysis, which evaluates selected time points of kinematic variables, SPM performs a statistical evaluation at each time point along the gait cycle, providing a continuous, frame-by-frame comparison [30,31].

SPM is based on random field theory (RFT)**,** which enables the detection of statistically significant deviations in smooth, time-dependent biomechanical data while controlling the family-wise error rate (FWER). The statistical approach used in this study was an SPM(t) test, analogous to a traditional *t*-test but applied to entire time series data. The calculated t-value curve across the gait cycle was compared to a “critical threshold (t)*”, determined through random field theory-based permutation tests. If the observed t-value curve exceeded t within a specific gait cycle region, that region was considered statistically significant [31].

Mathematically, SPM identifies statistically significant clusters in biomechanical waveforms using the following approach:

$$t\left(t\right)=\frac{\left\{{\bar{X}}_{1}-{\bar{X}}_{2}\right\}}{\sqrt{\frac{s_{1}^{2}}{n_{1}}+\frac{s_{2}^{2}}{n_{2}}}}$$

where

${\bar{X}}_{1},\;{\bar{X}}_{2}\;$are the means of the two groups (ITW vs. controls);

$s_{1}^{2},\;s_{2}^{2}$ are the variances of each group;

$n_{1},$ $n_{2}\;$are the sample sizes;

t(t) represents the statistical value computed at each time point along the GC.

The computed t-value curve was compared against a “critical threshold (t)*”, defined by

$$t*=max\left(\left|t(t)\right|\right)$$

where only regions where t(t) exceeds t* for a continuous duration are considered significant.

The significance level (α = 0.05) controls the probability of Type I error, ensuring that the findings represent true biomechanical differences rather than random noise.

## 3. Results

### 3.1. Spatio-Temporal Variables

Significant differences in spatio-temporal parameters were observed between children with ITW and the control group. The ITW group demonstrated a prolonged duration in both the single support phase (*p* < 0.02) and the swing phase (*p* < 0.02), while the control group exhibited significantly higher walking velocity (*p* = 0.014) compared to the ITW group. These findings reflect notable deviations in temporal gait dynamics and overall locomotor efficiency in children with ITW.

**Figure 1:**
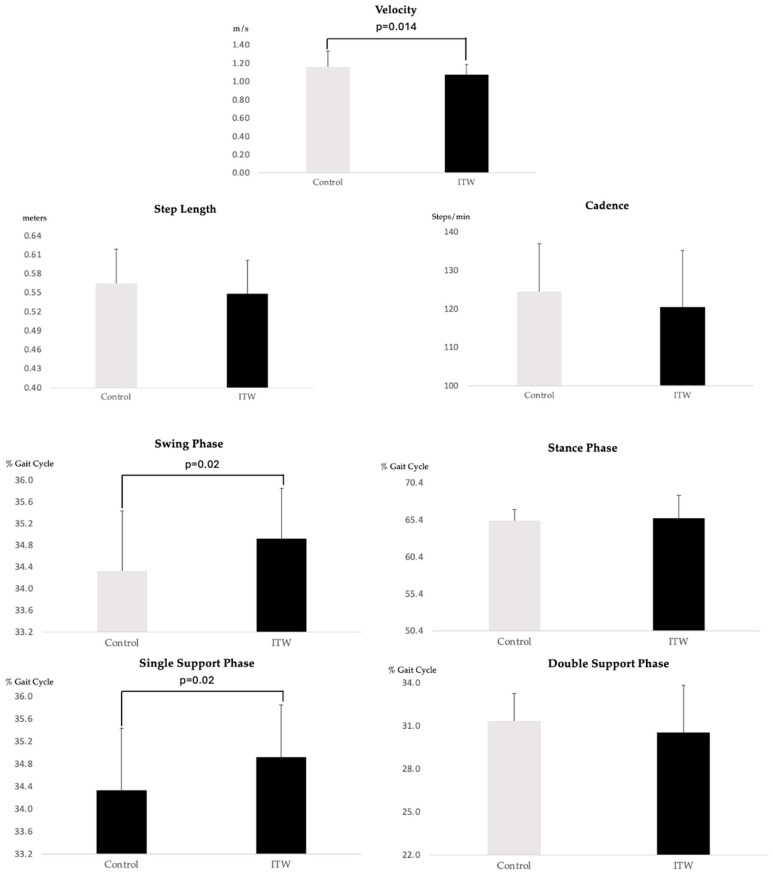
Spatio-temporal variables for ITW and control groups.

These results underscore the intricate adaptations of children with ITW and highlight the importance of temporal modifications in their gait patterns. Visual representation of these spatio-temporal findings is provided in Figure 1.

### 3.2. Sagittal Plane Kinematics: SPM Analysis

#### 3.2.1. Pelvis

**Figure 2:**
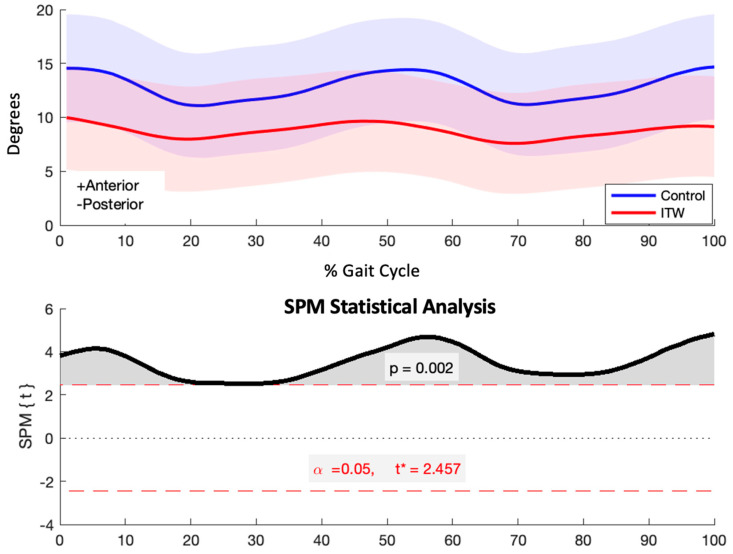
Sagittal plane pelvis kinematics (degrees) for control and ITW groups across the gait cycle (upper) and SPM analysis results (lower). The red dashed line indicates the critical threshold. The area of the *T*^2^ curve that crosses the critical threshold is shaded in grey and indicates the temporal location of significant kinematic differences.

The ITW group exhibited a significant reduction in anterior pelvic tilt throughout the gait cycle (*p* = 0.002), spanning both the stance and swing phases (Figure 2).

#### 3.2.2. Hip

**Figure 3:**
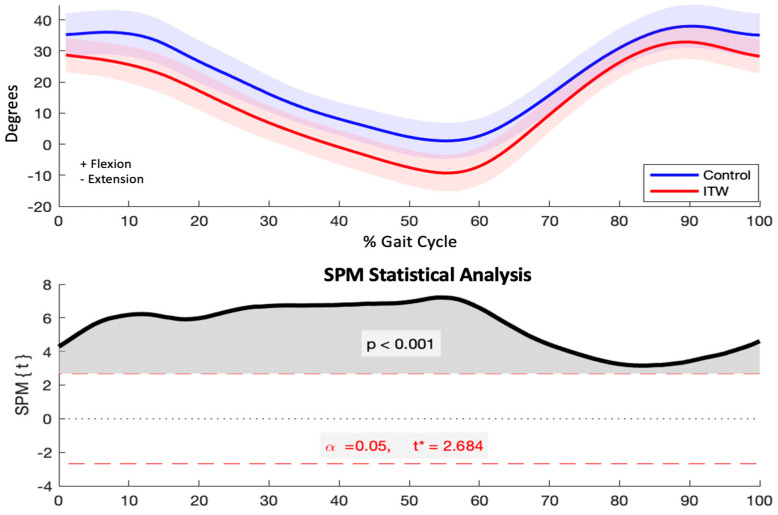
Sagittal plane hip kinematics (degrees) for control and ITW groups across the gait cycle (upper) and SPM analysis results (lower). The red dashed line indicates the critical threshold. The area of the *T*^2^ curve that crosses the critical threshold is shaded in grey and indicates the temporal location of significant kinematic differences.

Significant reductions in hip flexion angles were observed in the ITW group across the gait cycle (*p* < 0.001), encompassing both the stance phase (initial contact, loading response, mid-stance, and terminal stance) and the swing phase (pre-swing, initial swing, mid-swing, and terminal swing) (Figure 3).

#### 3.2.3. Knee

**Figure 4:**
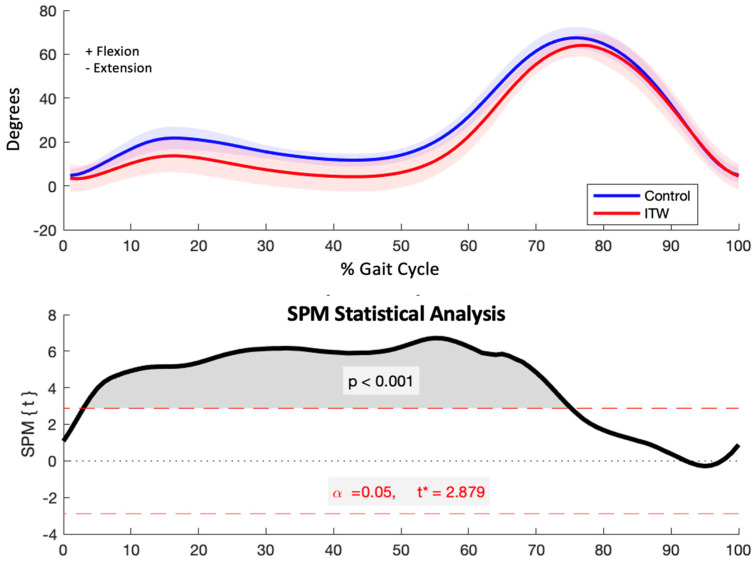
Sagittal plane knee kinematics (degrees) for control and ITW groups across the gait cycle (upper) and SPM analysis results (lower). The red dashed line indicates the critical threshold. The area of the *T*^2^ curve that crosses the critical threshold is shaded in grey and indicates the temporal location of significant kinematic differences.

The ITW group exhibited significantly reduced knee flexion during key phases of the gait cycle, from initial contact to pre-swing (*p* < 0.001). This encompasses critical phases such as initial contact, loading response, mid-stance, terminal stance, and pre-swing (Figure 4).

#### 3.2.4. Ankle

**Figure 5:**
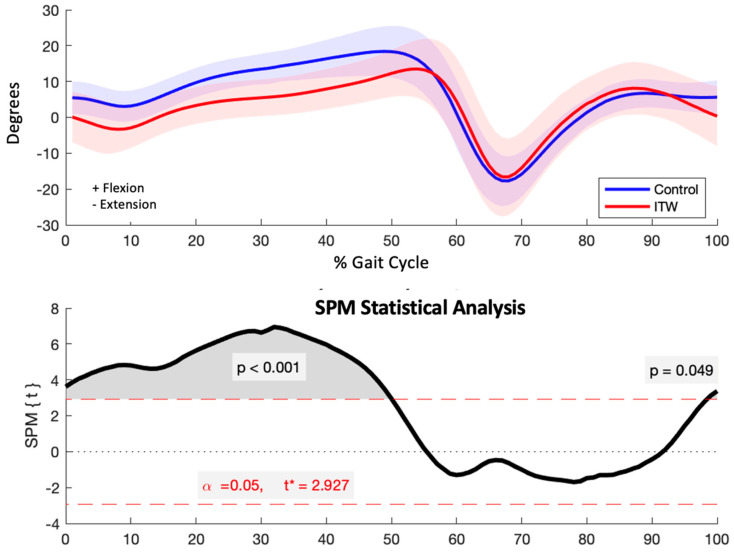
Sagittal plane ankle kinematics (degrees) for control and ITW groups across the gait cycle (upper) and SPM analysis results (lower). The red dashed line indicates the critical threshold. The area of the *T*^2^ curve that crosses the critical threshold is shaded in grey and indicates the temporal location of significant kinematic differences.

Ankle kinematics revealed significant increases in plantarflexion in the ITW group during both the stance and terminal swing phases. Excessive plantarflexion was observed from initial contact through the terminal stance (*p* < 0.001), phases typically requiring ankle dorsiflexion to facilitate smooth forward progression. Additionally, marked plantarflexion was detected during the terminal swing phase (*p* < 0.001), consistent with the characteristic toe-walking gait (Figure 5).

## 4. Discussion

This study provides a detailed evaluation of gait differences between children with ITW and typically developing children, focusing on spatio-temporal parameters and sagittal plane kinematics analyzed using SPM. The findings contribute valuable insights into the biomechanical adaptations and compensatory mechanisms underlying ITW gait, with implications for clinical assessment and intervention strategies.

### 4.1. Spatio-Temporal Adaptations

The spatio-temporal analysis revealed significant differences in walking speed, with the control group exhibiting a higher velocity compared to children with ITW (*p* = 0.014). This finding suggests that children with ITW may encounter limitations in generating and maintaining forward momentum, reflecting broader biomechanical constraints. These results contrast with previous studies that reported no significant differences in walking speed, cadence, or stride length and highlight the need to further explore the factors contributing to reduced velocity in this population [11,21]. The prolonged single support and swing phases observed in the ITW group suggest compensatory strategies aimed at optimizing stability and facilitating limb progression during critical phases of the gait cycle. In contrast, the reduced walking speed in this group may reflect a diminished capacity to generate and sustain forward propulsion, influenced by the combined effects of these temporal and kinematic adaptations.

Additionally, significant differences were observed in the single support and swing phases. The prolonged single support phase in the ITW group likely represents a compensatory strategy aimed at enhancing stability and facilitating efficient weight transfer during stance. Similarly, the extended swing phase may assist with limb advancement despite reduced ankle dorsiflexion, a hallmark of ITW gait. These temporal adaptations align with the findings of Westberry et al. [12], who identified timing adjustments as key compensatory mechanisms in ITW gait. Together, these results emphasize the intricate interplay of spatio-temporal adaptations employed by children with ITW to sustain basic gait functionality in the presence of underlying biomechanical challenges.

### 4.2. Sagittal Plane Kinematics

SPM analysis revealed key kinematic deviations in the sagittal plane. The significant reduction in anterior pelvic tilt throughout the gait cycle in the ITW group suggests altered postural control strategies aimed at maintaining stability. In contrast, the control group exhibited consistently greater anterior pelvic tilt during all phases of gait. This reduction in pelvic tilt in children with ITW likely represents a compensatory mechanism to counteract the forward momentum generated by excessive plantarflexion. These findings align with previous research emphasizing the role of pelvic adaptations in mitigating forward propulsion associated with excessive plantarflexion [36,37].

Additionally, the reduced hip flexion observed during both stance and swing phases suggests potential restrictions in hip range of motion or altered neuromuscular activation patterns. These limitations may impair gait efficiency, particularly during the swing phase, where greater hip flexion is crucial for effective limb advancement. To compensate for these deficits, children with ITW may adopt strategies such as increased cadence or prolonged swing duration. The interplay between these kinematic adaptations highlights the complex biomechanical compensations employed by children with ITW to maintain functional locomotion despite underlying gait deviations.

The reduced knee flexion observed during critical phases of the gait cycle (e.g., initial contact through pre-swing) underscores a stiffer, less dynamic gait pattern in children with ITW. This limitation likely increases muscular effort and alters joint loading, contributing to a less energy-efficient movement strategy. Such a pattern is characteristic of ITW and raises concerns about potential long-term musculoskeletal implications. Previous studies have similarly reported restricted knee flexion as a defining feature of ITW gait, often linked to increased joint stress and long-term musculoskeletal adaptations [21].

The pervasive plantarflexion observed during both stance and terminal swing phases further highlights the distinct ankle kinematics characteristic of ITW. Excessive plantarflexion during stance compromises the ankle rocker’s effectiveness, limiting forward progression and stability. The pronounced plantarflexion in terminal swing, although partially influenced by methodological considerations in gait event detection, underscores the persistent nature of ankle deviations in ITW and the need for targeted interventions. While methodological factors, such as the coordinate-based gait event detection algorithm described by Zeni et al. [36], may have contributed to the observed plantarflexion in terminal swing, the overall kinematic alterations at the ankle remain evident. These findings emphasize the necessity of robust event detection methodologies to improve the accuracy of kinematic assessments and optimize clinical interpretations.

The consistent reduction in anterior pelvic tilt observed throughout the gait cycle in the ITW group suggests a potential postural adaptation aimed at maintaining stability. By reducing forward tilt, children with ITW may shift their center of mass posteriorly, counteracting the forward momentum typically generated by increased plantarflexion, a hallmark of ITW gait. This adaptation may serve as a compensatory strategy to prevent forward falls, which aligns with findings from previous research on postural control differences in pediatric populations with gait abnormalities [12]. Similar adaptations have been reported in children with cerebral palsy and other gait disorders, where altered pelvic alignment compensates for reduced control at the ankle and hip joints [38,39]. Future investigations should explore the activation patterns of muscles responsible for pelvic control, such as the rectus abdominis and erector spinae, to elucidate the neuromuscular mechanisms underlying this altered strategy.

At the hip, the reduced flexion throughout both the stance and swing phases in children with ITW suggests potential limitations in hip range of motion or alterations in muscle activation patterns. This observation is consistent with previous studies reporting hip flexion deficits in children with ITW, potentially limiting step length and impacting the efficiency of the swing phase [11,40]. Reduced hip flexion has also been associated with compensatory strategies such as increased cadence to maintain walking speeds comparable to typically developing peers [41]. Further research is needed to explore whether these deficits are linked to specific muscular restrictions, such as tightness in the rectus femoris or psoas, or if they arise from coordination deficits between the pelvis and femur.

The significantly reduced knee flexion observed in the ITW group, particularly during the weight acceptance and mid-stance phases, points to a less dynamic and potentially less energy-efficient gait pattern. This finding aligns with previous research that highlighted altered joint loading and increased muscular effort in children with ITW [42,43]. A stiffer gait pattern at the knee joint may increase the metabolic cost of walking, as reported in studies on gait abnormalities where reduced knee flexion limits shock absorption and energy transfer [44,45]. Longitudinal studies are warranted to determine whether this pattern contributes to long-term musculoskeletal adaptations or predisposes these children to joint pathologies in adulthood.

The analysis of ankle kinematics revealed significant and persistent plantarflexion throughout the gait cycle in the ITW group, particularly during the early stance phases (0–55%) and again at the end of the gait cycle (98–100%). While this latter observation may be attributed to the limitations of the coordinate-based gait event detection algorithm used in this study, as described by Zeni et al. [36], it underscores the pervasive nature of altered ankle kinematics in children with ITW. These findings are consistent with the defining characteristics of ITW, where increased plantarflexion compensates for restricted dorsiflexion, often exacerbated by shortened triceps surae musculature [11,12]. The interpretation of kinematic data requires careful consideration of methodological factors, such as the absence of force plate data, which may influence the timing and displacement of gait curves.

These findings collectively emphasize the need for a nuanced understanding of the biomechanical adaptations in ITW, particularly as they relate to long-term musculoskeletal health and functional outcomes. Advanced analytical techniques like SPM have proven instrumental in detecting subtle yet clinically significant differences in gait patterns. Future research should aim to elucidate the long-term consequences of these deviations, particularly their potential impact on secondary musculoskeletal complications, and to refine intervention strategies to optimize gait function and quality of life in children with ITW. Additionally, the role of computational modeling and refined event detection algorithms should be explored to improve the precision and applicability of gait analyses in both clinical and research settings.

## 5. Conclusions

This study provides robust evidence of distinct gait deviations in children with Idiopathic Toe Walking, particularly in the sagittal plane kinematics of the pelvis, hip, knee, and ankle joints. While spatio-temporal variables remained largely comparable between children with ITW and their typically developing peers, significant kinematic differences underscore the altered neuromuscular control and joint mechanics in this population. Specifically, reduced anterior pelvic tilt, limited hip flexion, decreased knee flexion, and excessive plantarflexion reflect the complex biomechanical adaptations underlying the ITW gait pattern. These findings highlight the critical need for targeted biomechanical assessments and interventions to address these deviations and mitigate their potential long-term consequences.

Comprehensive clinical assessments that incorporate advanced kinematic analyses, such as SPM, are essential for detecting subtle but clinically significant gait deviations. These analyses provide valuable insights into the specific biomechanical deficits associated with ITW, facilitating the development of individualized interventions aimed at restoring functional joint mechanics. Early therapeutic strategies should prioritize improving hip and knee flexion during gait, enhancing ankle dorsiflexion, and correcting altered pelvic mechanics. Such approaches could prevent the progression of secondary musculoskeletal complications and improve overall functional outcomes.

Future research should focus on longitudinal studies to elucidate the natural progression of ITW and its impact on musculoskeletal development over time. Additionally, investigating the efficacy of various intervention strategies—including physical therapy, orthotic devices, and, when necessary, surgical procedures—will be vital in establishing evidence-based guidelines for the management of ITW. Advances in gait analysis technology, including the refinement of event detection algorithms and the integration of wearable motion sensors, hold promise for improving the precision and applicability of kinematic data in both clinical and research settings.

Furthermore, the differences in single support and swing phase durations observed between the ITW and control groups suggest an altered temporal distribution of gait phases that warrants further exploration. Future studies should examine the implications of these temporal changes on joint loading, energy expenditure, and overall gait efficiency. Expanding research to include larger, more diverse populations will enhance the generalizability of findings and contribute to a more comprehensive understanding of ITW.

In conclusion, while children with ITW may superficially maintain gait patterns resembling those of typically developing peers, the underlying kinematic abnormalities necessitate early, targeted interventions. Addressing these biomechanical challenges proactively can significantly enhance gait function, reduce the risk of secondary complications, and improve the quality of life for affected children. Collaboration between clinicians and researchers in advancing both assessment techniques and therapeutic approaches will be pivotal in achieving optimal outcomes for this population.

## Data Availability

The data presented in this study are available on request from the corresponding author. The data are not publicly available due to ethical and legal restrictions, as they contain sensitive patient information collected in a clinical setting. In accordance with data protection regulations and hospital policies, access to these data is restricted to ensure patient confidentiality and compliance with institutional guidelines.

## Abbreviations

The following abbreviations are used in this manuscript:

ITW	Idiopathic Toe Walking
TD	Typically Developing
SPM	Statistical Parametric Mapping
ROM	Range of Motion
EMG	Electromyography
GC	Gait Cycle
3D	Three-Dimensional
C	Control Group
